# The effect of detoxification on acoustic features of Mandarin speech in male heroin users

**DOI:** 10.1371/journal.pone.0304399

**Published:** 2024-06-12

**Authors:** Puyang Geng, Ningxue Fan, Zhijun Li, Rong Ling, Kai Yang, Xiao Mao, Hong Guo

**Affiliations:** 1 Department of Audio, Video, and Electronic Forensics, Academy of Forensic Science, Shanghai, China; 2 Shanghai Forensic Service Platform, Key Laboratory of Forensic Science, Ministry of Justice, Shanghai, China; 3 Information Security and Social Management Innovation Lab, Shanghai Open University, Shanghai, China; University of Missouri Columbia, UNITED STATES

## Abstract

This study aims to investigate the effect of detoxification on acoustic features of Mandarin speech. Speech recordings were collected from 66 male abstinent heroin users with different durations of drug detoxification, specifically early abstinent users with a detoxification duration of less than 2 years, sustained abstinent users with 2 years of detoxification, and long-term abstinent users with a detoxification duration of more than 2 years. The results of the acoustic analyses showed that early abstinent users exhibited lower loudness, relative energies of F1, F2, and F3, higher H1–A3, and fewer loudness peaks per second, as well as a longer average duration of unvoiced segments, compared to the sustained and long-term abstinent users. The findings suggest that detoxification may lead to a rehabilitation process in the speech production of abstinent heroin users (e.g., less vocal hoarseness). This study not only provides valuable insights into the effect of detoxification on speech production but also provides a theoretical basis for the speech rehabilitation and detoxification treatment of heroin users.

## 1. Introduction

Drug (e.g., heroin, cocaine, marijuana, and methamphetamine) abuse can result in significant organ damage, thereby exerting a detrimental impact on the health status of drug users. In the process of detoxification, it is commonly anticipated that drug users would be able to reduce or cease drug consumption and restore their health. Nevertheless, research has revealed that detoxification may not necessarily engender a change in the health condition of drug users; in fact, it may even precipitate more severe complications [1, 2].

At present, there is a growing focus on the rehabilitation and reintegration of drug users into normal social life. As a fundamental instrument for social interaction, speech production holds significant importance in facilitating successful interpersonal communication and fostering positive social relationships [3]. However, a considerable body of research has reported significant differences in acoustic features of speech between drug users and non-drug users.

Previous studies on American English speakers have reported that both heroin and cocaine use would lead to decreased vocal control and dysphonia [4–6]. Besides, some evidence from acoustic analyses has also demonstrated that 3,4-methylamphetamine (MDMA; also known as ecstasy) users showed a higher second formant (i.e., F2), and lower F2 variability and fundamental frequency (henceforth, F0) [7]. Further, a classification analysis of MDMA, oxytocin, and placebo users indicates that a promising accuracy (i.e., 92%) could be achieved based on acoustic parameters, such as F0, pause, and MFCCs (Mel-frequency cepstral coefficients) [8]. Additionally, Agurto and her colleagues applied similar research paradigm on cocaine users, the results showed no significant acoustic difference (e.g., pitch variation, vowel space feature, voice quality, and MFCC) between the abstinence and current cocaine users [9]. However, they observed a relatively good performance in classification tasks, with an accuracy of over 80% in predicting cocaine abstinence using linguistic features (e.g., acoustic and semantic features) [9, 10]. Another automatic classification analysis on cannabis-intoxication speech, consistent with previous studies, reported encouraging model performance (around 69%) in predicting cannabis intoxication using mel-spectrograms from sustained vowels [11]. Previous study conducted on Mandarin Chinese speakers exhibited that the users of certain drugs (i.e., heroin, ketamine, and methamphetamine) showed smaller F0 standard deviation, reduced loudness, and cepstral peak prominence, as well as higher H1–A3, longer unvoiced segments, and fewer voiced segments per second [12]. Therefore, some scholars have proposed that these differences indicate that the acoustic characteristics of drug users deviate from the norm [12, 13].

Additionally, the impact of alcohol on speech production has been widely studied, and previous research has yielded varying results. Early studies suggested that abstinent alcoholics showed no significant incongruencies in speech patterns [14]. However, studies on reading speech in alcoholics during sober and intoxicated states have found that intoxicated individuals tend to exhibit more speech errors, such as longer reading time, increased word omission, and revision [15]. In recent years, research on alcohol intoxication speech has found significant differences in the mean and range of F0 between abstinent alcoholics and healthy controls during the expression of emotional speech [16]. From the perspective of automatic detection, Schuller et al. [17] reviewed previous alcohol intoxication state classification challenges at the INTERSPEECH conference, outlining common features (e.g., low-level descriptors extracted by OpenSMILE, including energy, F0, and MFCC) and algorithms (e.g., SVM, KNN, and GMM) used in these challenges. Their research reported the detection accuracy for alcohol intoxication can reach approximately 70% and emphasized the necessity of speaker-independent splitting when performing speaker state detection tasks. More recent research using acoustic features and various classifiers (e.g., SVM, Random Forest, KNN) has achieved recognition accuracies of around 80% for distinguishing between alcohol and non-alcohol states [18]. Similarly, support vector machine model exhibited high accuracy (i.e., 98%) in predicting alcohol intoxication using spectrogram features (e.g., MFCC) [19].

Scholars have engaged in discussions regarding the reasons behind the occurrence of acoustic changes in drug users. It has been reported that medicinal users of cannabis exhibit a significant increase in the voice onset time (VOT) during the production of bilabial sounds (e.g., /b/, /p/) and alveolar sounds (e.g., /d/, /t/), indicating an extended control time over the vocal tract and lips. Moreover, there is a larger shimmer, suggesting a decline in the control force of the vocal folds [6]. Indeed, numerous studies on drug users have consistently documented that drug use can cause significant damage to the speech organs. Consensus has been reached among researchers that marijuana, methamphetamine, ketamine, and opioid (e.g., cocaine, heroin) drugs can lead to respiratory depression in drug users [20–22]. In addition, studies have found that heroin and cocaine can cause palatal perforation and nasal septal perforation [23–25]. There have been case reports suggesting that marijuana and opioid drugs may lead to vocal nodules or vocal cord paralysis [20, 26]. Aides et al. [27] and Marco et al. [28] discovered that ketamine can result in tongue movement disorders, characterized by sustained lingual contraction.

It is then reasonable to ask whether the speech production of drug users tend to normalize with detoxification. However, there is a paucity of research on the acoustic changes in drug users after detoxification. Some scholars have hypothesized that cognitive dysfunction in drug users gradually normalizes along with the detoxification process [29]. Similarly, other scholars have suggested that the cognitive dysfunctions in drug users would gradually recover after detoxification [30]. On the contrary, most studies have held opposite opinions that those dysfunctions do not alleviate with increasing abstinence duration, and significant differences still exist between drug users and healthy controls [31, 32]. Therefore, whether the acoustic changes of drug users can gradually recover during the detoxification process is also a question worthy of attention.

The current study was then set out to investigate the effect of drug detoxification on acoustic features of speech in drug users. Considering that heroin is one of the most prevalent drugs of abuse in China [33], the abstinent heroin users with different durations of detoxification (i.e., the last drug use was within a period of less than 2 years, 2 years, and longer than 2 years) were involved in the current study. Speech recordings were then collected from sixty-six male abstinent heroin users, twenty-two of whom have been in drug detoxification for less than 2 years, thirty-seven have been in drug detoxification for 2 years, and seven have been in drug detoxification for longer than 2 years. Acoustic analysis was performed to reveal the difference of speech production across the three groups of abstinent heroin users. This paper seeks to understand the effect of detoxification on speech production and provide theoretical foundation for the speech rehabilitation and detoxification treatment of drug users.

## 2. Methods

This research was approved by the Committee for the Protection of Human Subjects (CPHS) at the Academy of Forensic Science (Shanghai, China). All subjects involved in the current study were recruited to participate in this experiment between July and September 2022 and had indicated their awareness of the research purpose and voluntarily participated in the experiment. The written consent forms have obtained from all participants.

### 2.1 Speech recording

Sixty-six male heroin users aged from 26–53 were recruited from a compulsory drug rehabilitation centre in China, among which twenty-two have been in drug abstinence for less than two years (nineteen were in the abstinence for less than two month), thirty-seven have been in abstinence for two years, and seven have been in abstinence for longer than 2 years (a criterion suggested by Huang et al. [29]). In terms of narrative convenience, the first group of subjects will be referred to as early abstinent heroin users (< 2 years), the second group as sustained abstinent heroin users (2 years), and the third group as long-term abstinent heroin users (> 2 years). The average durations of previous heroin usage were 9.0 years, 4.4 years, and 7.4 years for the early, sustained, and long-term abstinent users, respectively. All heroin users were native speaker of Mandarin Chinese and spoke fluent standard Mandarin.

**Fig 1:**
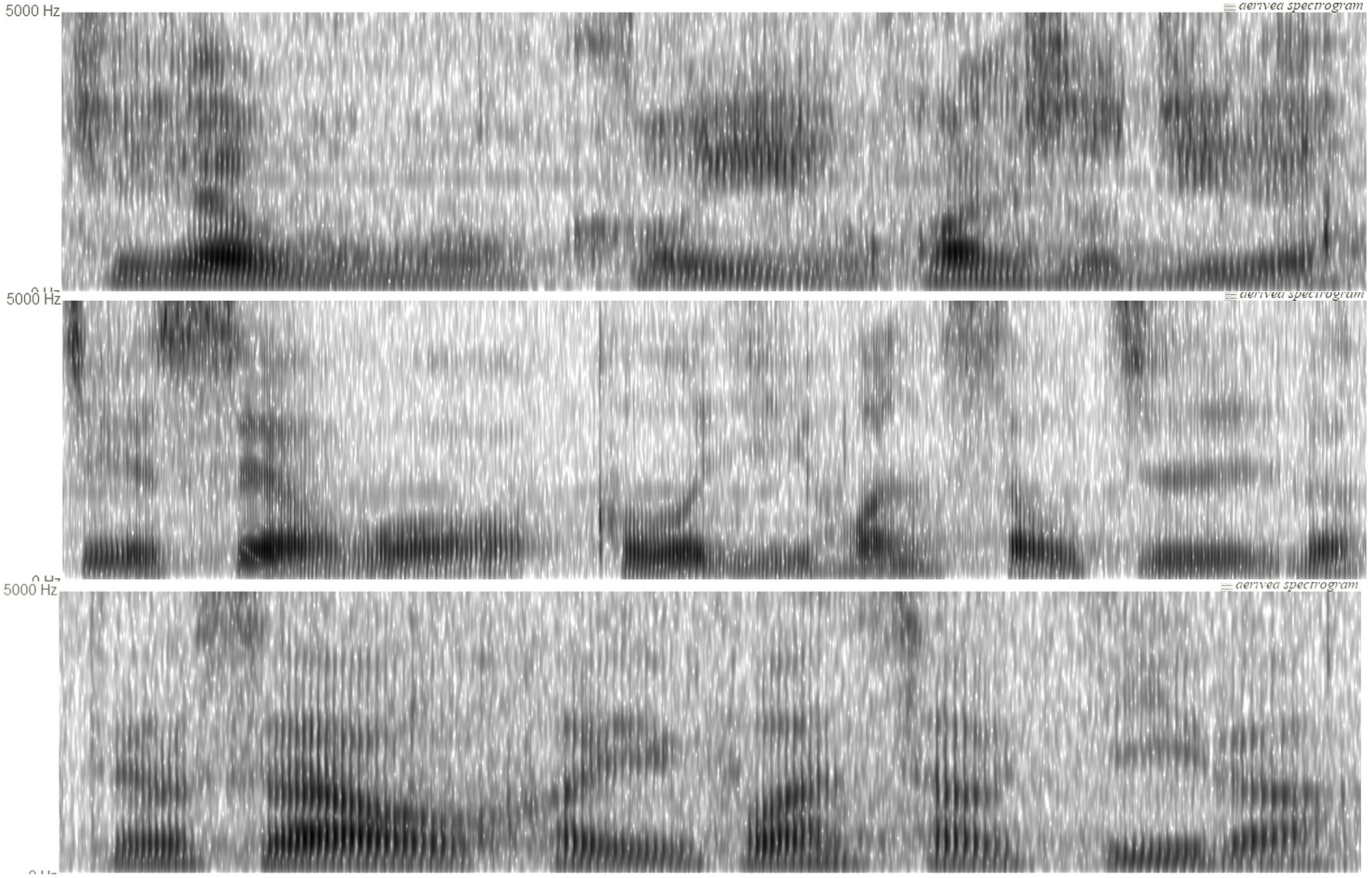
The spectrogram (from 0 to 5000 Hz) of an example speech (i.e., Sentence 26: /ʈʂɤ4/ /ɕja4/ /uɔ3/ /kʰɤ3/ /i3/ /xwan4/ /ʂoʊ3/ /tɕi1/ /lɤ0/) produced by of the early (bottom), sustained (middle), and long-term (top) abstinent heroin users.

As shown in S1 Appendix, twenty-six phonetically balanced target sentences were designed for the current study. Before the experiment, all participants were first asked to familiarize the speech materials. Speech recording experiment was conducted in a sound-attenuated room. The unidirectional lavalier microphone was placed about 25 cm away from the speaker’s mouth. The audio was recorded using a portable high-quality digital recorder (Zoom H5n) with a sample rate was set to 44.1 kHz and 16 bits resolution. All participants were instructed to read aloud the target sentences as per the sequence prescribed in S1 Appendix using their normal speech style. In the event of mispronunciation, the sentence is reiterated until correctly delivered. Altogether, 26 (target sentences) * 66 (speakers) = 1716 recordings were collected for the current research. The average duration of each speech recording is around 1.7 seconds, with a range of 0.6 to 7.2 seconds. The total duration of all recordings is around 0.8 hour. The spectrograms of an example speech produced by of the early, sustained, and long-term abstinent heroin users were shown in Fig 1.

### 2.2 Data extraction

The Geneva Minimalistic Parameter Set (GeMAPS) [34, 35] is a well-established and standard acoustic feature set, which has been widely used in previous studies on pathological speech (e.g., [36, 37]). Hence, the GeMAPS acoustic features were automatically extracted for all collected speech using openSmile (i.e., a standard audio feature extraction tool) in Python 3.8.10. A detailed description and implementation of these feature set is given in [35], The acoustic features analysed in the current study are classified into four categories, viz., frequency-related feature, amplitude-related feature, spectral-related feature, and temporal-related feature.

#### Frequency-related feature

The mean, standard deviation, and range of logarithmic fundamental frequency (F0) on a semitone scale, starting at 27.5 Hz (semitone 0; please note that as 0 is reserved for unvoiced frames, every value below semitone 1 [29.136 Hz] is clipped to 1); jitter (i.e., the cycle-to-cycle variation of F0; for the formula used to calculate jitter see Eyben et al. [35]); mean of the first, second and third formants’ (i.e., F1, F2, F3) centre frequency in Hertz.

#### Amplitude-related feature

Shimmer (i.e., the measures of the difference in amplitude from cycle to cycle; for the formula used to calculate shimmer see Eyben et al. [35]), loudness (i.e., estimate of perceived signal intensity from an auditory spectrum), and Harmonic-to-Noise Ratio (i.e., HNR; relation of energy in harmonic components to energy in noise-like components).

#### Spectral-related feature

The relative energies of F1, F2, and F3 (The ratio of the energy of the spectral harmonic peak at the formant’s centre frequency to the energy of the spectral peak at F0), H1-H2 (i.e., the difference between the amplitudes of the first harmonic and the second in the Fourier spectrum), and H1-A3 (i.e., the difference between the amplitudes of H1 and the amplitude of F3).

#### Temporal-related feature

Loudness-peaks per second (i.e., number of loudness peaks per second), voiced-segments mean (i.e., average duration of voiced segments), unvoiced-segments mean (i.e., average duration of unvoiced segments), and voiced-segments per second (i.e., number of continuous voiced regions per second).

### 2.3 Statistical analysis

All nineteen features (i.e., frequency-related feature, amplitude-related feature, spectral-related feature, and temporal-related feature) were statistically analysed to investigate the acoustic difference across the three groups of speakers (i.e., the early, sustained, and long-term abstinent heroin users). Linear-mixed effect models were built for the nineteen acoustic features using *lme4* and *lmerTest* packages in R software [38, 39]. In each model, the duration of drug detoxification (i.e., the early, sustained, and long-term abstinent heroin users) was fixed effect, and the intercepts for speaker and target sentence were random effects. Tukey HSD post-hoc tests were then conducted on all significant effects for more detailed analyses using *lsmeans* package [40].

## 3. Results

**Fig 2:**
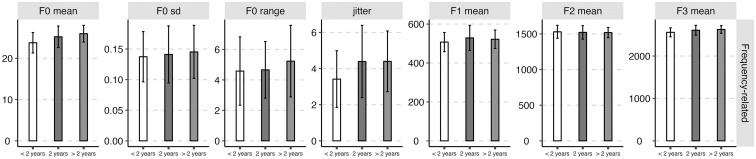
The mean value (standard deviation as error bars) of the frequency-related acoustic parameters of the early (i.e., < 2 years), sustained (i.e., 2 years), and long-term abstinent heroin users (i.e., > 2 years).

**Fig 3:**
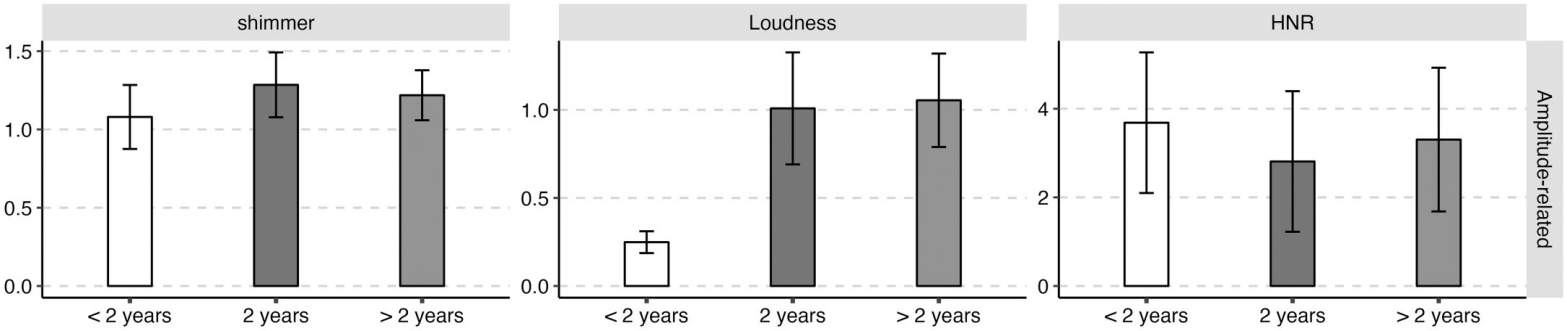
The mean value (standard deviation as error bars) of the amplitude-related acoustic parameters of the early (i.e., < 2 years), sustained (i.e., 2 years), and long-term abstinent heroin users (i.e., > 2 years).

**Fig 4:**
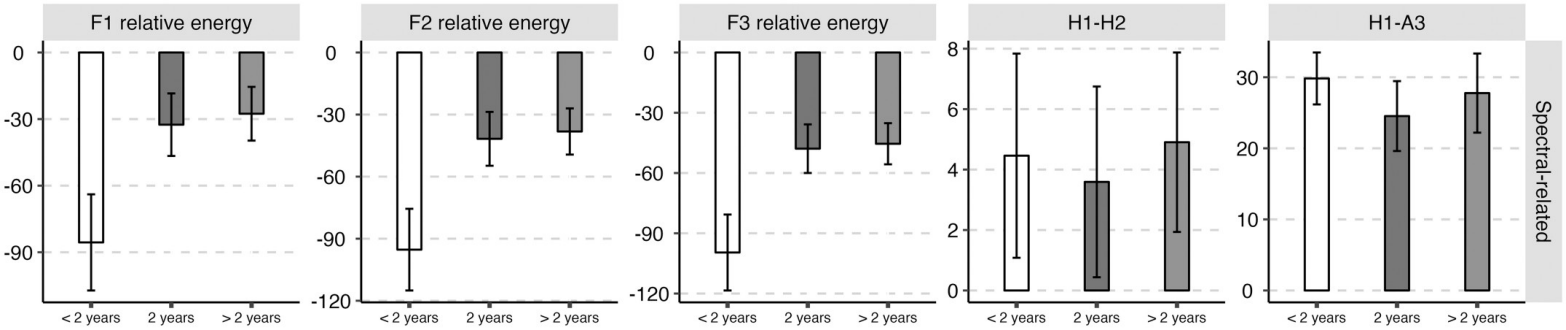
The mean value (standard deviation as error bars) of the spectral-related acoustic parameters of the early (i.e., < 2 years), sustained (i.e., 2 years), and long-term abstinent heroin users (i.e., > 2 years).

**Fig 5:**
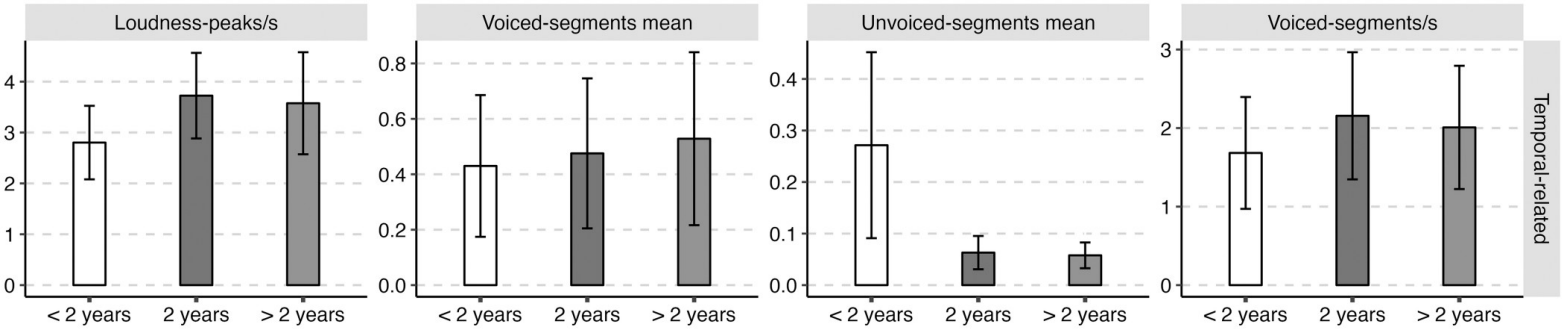
The mean value (standard deviation as error bars) of the temporal-related acoustic parameters of the early (i.e., < 2 years), sustained (i.e., 2 years), and long-term abstinent heroin users (i.e., > 2 years).

The average values and standard deviations of the nineteen acoustic features of the three groups of abstinent heroin users (i.e., the early, sustained, and long-term abstinent users) are presented in Table 1. The mean values (standard deviations as error bars) of the four categories of acoustic features (i.e., frequency-related feature, amplitude-related feature, spectral-related feature, and temporal-related feature) for the three groups of abstinent heroin users (i.e., the early, sustained, and long-term abstinent users) were plotted in Figs 2–5.

**Table 1:** The mean values (standard deviations) of the nineteen acoustic features of the three groups of abstinent heroin users (i.e., the early, sustained, and long-term abstinent users).

Acoustic features	Duration of drug detoxification		
	Early abstinent users	Sustained abstinent users	Long-term abstinent users
F0 mean	23.77(2.48)	25.22(2.6)	25.97(2.03)
F0 sd	0.14(0.04)	0.14(0.05)	0.15(0.04)
F0 range	4.58(2.24)	4.66(1.86)	5.23(2.35)
jitter	3.42(1.57)	4.39(2)	4.4(1.68)
F1 mean	507.33(49.33)	528.97(64.42)	521.96(47.26)
F2 mean	1529.56(90.78)	1521.86(95.01)	1519.02(71.99)
F3 mean	2558.73(106.08)	2608.58(114.99)	2626.74(90.43)
loudness	0.25(0.06)	1.01(0.32)	1.05(0.27)
shimmer	1.08(0.2)	1.28(0.21)	1.22(0.16)
HNR	3.68(1.59)	2.81(1.59)	3.3(1.62)
F1 relative energy	-85.57(21.66)	-32.52(14.09)	-27.58(12.11)
F2 relative energy	-95.29(19.74)	-41.72(13.02)	-38.18(11.15)
F3 relative energy	-99.56(18.9)	-47.88(12.1)	-45.43(10.25)
H1-H2	4.46(3.38)	3.59(3.16)	4.91(2.97)
H1-A3	29.83(3.65)	24.54(4.92)	27.77(5.56)
Loudness-peaks/second	2.8(0.72)	3.72(0.84)	3.58(1)
Voiced segments mean	0.43(0.26)	0.48(0.27)	0.53(0.31)
Unvoiced segments mean	0.27(0.18)	0.06(0.03)	0.06(0.03)
Voiced-segments/second	1.68(0.71)	2.16(0.81)	2.01(0.78)

The results of linear-mixed effect models on the nineteen acoustic features were presented in Table 2. For frequency-related features, significant main effects of “the duration of drug detoxification” were observed for F0 mean and jitter. Tukey HSD post hoc tests were then conducted, and the results showed that early abstinent users exhibited lower jitter as compared to the other two groups of abstinent users (Early-Sustained: *β* = -0.98, *SE* = 0.25, *z* = -3.86, *p* < 0.001; Early-Long-term: *β* = -0.99, *SE* = 0.41, *z* = -2.42, *p* = 0.048).

**Table 2:** The results of linear-mixed effect models on the nineteen acoustic features. In each model, the duration of drug detoxification (i.e., the early [< 2 years], sustained [= 2 years], and long-term [> 2 years] abstinent heroin users) was fixed effect.

Acoustic features		Models			Post hoc tests
		*df*	*F*	*p*	Tukey HSD
Frequency-related feature	F0 mean	63	3.48	**0.04**	/
	F0 sd	63	0.34	0.71	
	F0 range	63	1.05	0.36	
	Jitter	63	7.96	**<0.001**	Early < Sustained; Early < Long-term
	F1 mean	63	1.82	0.17	/
	F2 mean	63	0.12	0.89	
	F3 mean	63	2.86	0.07	
Amplitude-related feature	Shimmer	63	17.16	**<0.001**	Early < Sustained; Early < Long-term
	Loudness	63	72.78	**<0.001**	Early < Sustained; Early < Long-term
	HNR	63	2.94	0.06	/
Spectral-related feature	F1 relative energy	63	251.23	**<0.001**	Early < Sustained; Early < Long-term
	F2 relative energy	63	304.59	**<0.001**	Early < Sustained; Early < Long-term
	F3 relative energy	63	311.99	**<0.001**	Early < Sustained; Early < Long-term
	H1-H2	63	1.36	0.27	/
	H1-A3	63	12.58	**<0.001**	Early > Sustained
Temporal-related feature	Loudness-peaks per second	63	30.39	**<0.001**	Early < Sustained; Early < Long-term
	Voiced segments mean	63	2.27	0.11	/
	Unvoiced segments mean	63	328.94	**<0.001**	Early > Sustained; Early > Long-term
	Voiced segments per second	63	13.72	**<0.001**	Early < Sustained

For amplitude-related features, significant main effects of “the duration of drug detoxification” were observed for shimmer and loudness. The results of post hoc test showed that early abstinent users exhibited lower loudness (Early-Sustained: *β* = -0.76, *SE* = 0.07, *z* = -11.58, *p* < 0.001; Early-Long-term: *β* = -0.81, *SE* = 0.11, *z* = -7.62, *p* < 0.001) and shimmer (Early-Sustained: *β* = -0.21, *SE* = 0.04, *z* = -5.86, *p* < 0.001; Early-Long-term: *β* = -0.14, *SE* = 0.06, *z* = -2.46, *p* = 0.04) than the sustained and long-term abstinent users.

Significant main effects of “the duration of drug detoxification” were observed for all spectral-related feature except for H1-H2. Tukey HSD post hoc analyses showed that early abstinent heroin users exhibited lower relative energies of F1 (Early-Sustained: *β* = -53.05, *SE* = 2.48, *z* = -21.39, *p* < 0.001; Early-Long-term: *β* = -57.98, *SE* = 4.00, *z* = -14.50, *p* < 0.001), F2 (Early-Sustained: *β* = -53.58, *SE* = 2.31, *z* = -23.21, *p* < 0.001; Early-Long-term: *β* = -57.11, *SE* = 3.72, *z* = -15.35, *p* < 0.001), and F3 (Early-Sustained: *β* = -51.68, *SE* = 2.15, *z* = -24.04, *p* < 0.001; Early-Long-term: *β* = -54.14, *SE* = 3.47, *z* = -15.62, *p* < 0.001) than the other two groups of abstinent users; and (2) higher H1-A3 than the sustained abstinent users (Early-Sustained: *β* = 5.29, *SE* = 1.07, *z* = 4.95, *p* < 0.001).

Significant main effects of “the duration of drug detoxification” were found for all temporal-related features, with the only exception of voiced-segments mean. The results of post hoc analysis showed that early abstinent users exhibited fewer loudness peaks per second (Early-Sustained: *β* = -0.92, *SE* = 0.12, *z* = -7.72, *p* < 0.001; Early-Long-term: *β* = -0.77, *SE* = 0.19, *z* = -4.01, *p* < 0.001) and longer average duration of unvoiced segments (Early-Sustained: *β* = 0.21, *SE* = 0.01, *z* = 24.77, *p* < 0.001; Early-Long-term: *β* = 0.21, *SE* = 0.01, *z* = 15.76, *p* < 0.001) than the sustained and long-term abstinent users. Besides, early abstinent users showed fewer voiced segments per second as compared to the sustained abstinent users (Early-Sustained: *β* = -0.47, *SE* = 0.09, *z* = -5.23, *p* < 0.001).

## 4. Discussion

The current study investigated the speech pattern of 66 heroin users with different durations of drug detoxification (i.e., 22 early abstinent users [< 2 years], 37 sustained abstinent users [= 2 years], and 7 long-term abstinent users [> 2 years]). The results of acoustic analysis showed significant effects of detoxification on the amplitude-related (i.e., shimmer and loudness), spectral-related (i.e., relative energies of F1, F2, and F3, and H1-A3), and temporal-related features (i.e., loudness peaks and voiced segments per second, and average duration of unvoiced segments).

Previous research has established that higher H1-A3 indicate the presence of hyper-functional voice disorders (i.e., vocal hoarseness) in speakers. It has found that the previous heroin, ketamine, and methamphetamine users exhibit higher H1-A3 compared to healthy control groups, indicating the presence of vocal hoarseness in drug users [12]. Therefore, the most significant finding observed in the present study is that the sustained and long-term abstinent heroin users demonstrated reduced H1-A3, indicating an alleviation of vocal hoarseness in the two groups of abstinent users. It is difficult to explain this result without further medical examinations on these drug users, but there are several possible explanations for this result. As the duration of detoxification increases, the respiratory function of drug users may improve, leading to more stable airflow during speech production. Alternatively, the tension in the vocal fold muscles of drug users may increase, enhancing control over the vocal folds. These explanations require future specialized physiological research to validate.

In addition, the increasing vocal intensity (e.g., loudness, relative energies of F1, F2, and F3, voiced segments, and loudness peaks per second) observed for the sustained and long-term abstinent heroin users may suggest an enhancement in the spectral energy of their speech spectrum, as well as an increased prevalence of spectral harmonic components. Comparing with previous acoustic measurements on healthy non-drug users (i.e., H1-A3 = 23.12, loudness = 1.32, F1 relative energy = -31.62, F2 relative energy = -40.48, F3 relative energy = -45.64, and voiced segments per second = 2.23) [12], the above findings in the current paper collectively support the hypothesis that detoxification may lead to a gradual recovery in the speech production of abstinent heroin users.

From Table 1, a further comparison among the three groups of abstinent heroin users shows an ascending order of F0 measurements (i.e., mean, standard deviation, and range), i.e., early < sustained < long-term. Although it is not rigorous here to make a direct comparison across groups of abstinent users, we can still see the distinction roughly. In specific, the longer the duration of detoxification, the greater the variation in fundamental frequency observed in heroin users. In other words, as abstinent heroin users undergo detoxification, their control over the vocal folds tends to approach that of healthy controls (the measurements of the healthy non-drug users from Geng et al. [12] are taken as a reference: F0 mean = 25.29, F0 sd = 0.18, and F0 range = 4.77).

## 5. Limitations

Several limitations of the present study should also be noted. In the first place, only seven long-term abstinent users (i.e., > 2 years) were recruited in the current study. The small sample size of the long-term abstinent users may not be representative of the larger population of long-term abstinent users. Therefore, the results should be interpreted with caution and may not be generalizable to all long-term abstinent users. The predominance of short-term abstinent users may also introduce limitations in result interpretation. Our findings may primarily reflect the effects of detoxification on this specific stage, rather than providing a comprehensive understanding of the entire recovery process. To improve future studies, it is crucial to increase the sample size of the long-term abstinent users and balance the sample size of each group to enhance the reliability and validity of the findings. Additionally, the lack of female participants in our study should be acknowledged as a limitation. By not including female participants, our findings may not be representative of the entire population and may overlook potential gender-related differences in the effects of detoxification. Further studies involving female participants will enable a more comprehensive understanding of the effects of detoxification on the speech production. Thirdly, a longitudinal study on all abstinent drug users is of utmost necessity to further corroborate the findings of this research. Lastly, an investigation of the effect of different drug types (e.g., ketamine, Methamphetamine, etc.) will be necessary to draw a full picture of the effect of detoxification on speech production.

## 6. Conclusion

This study set out to investigate the effect of detoxification on speech production. The current study revealed that, taken heroin as an example, significant changes were observed for amplitude-related, spectral-related, and temporal related feature, which correlated with the extended duration of drug detoxification. In general, it seems drug detoxification might result in a speech rehabilitation (e.g., less vocal hoarseness) of the abstinent heroin user. Not only does this study enhance the understanding of the impact of drug use on speech production, but it also provides a theoretical basis for the speech rehabilitation and detoxification treatment of drug users.

## Supporting information

S1 Appendix Target sentences designed in the current study. (DOCX)

S1 Checklist Human participants research checklist. (DOCX)

S1 Data (CSV)

S2 Data (CSV)
