# Supplementary material for: The effect of detoxification on acoustic features of Mandarin speech in male heroin users
Source: PLoS One. 2024 Jun 12;19(6):e0304399. doi: 10.1371/journal.pone.0304399 (PMC11168614; doi:10.1371/journal.pone.0304399)
Supplement: S1 Appendix — (DOCX) [file pone.0304399.s001.docx]

**Appendix 1.** Target sentences designed in the current study.

| **No.** | **Target sentences in Chinese pinyin** |
| --- | --- |
| 1 | /ʈʂɤ4/ /li3/ /pu4/ /nɤŋ2/ /ɕi1/ /jɛn1/ |
| 2 | /ni3/ /mən0/ /tsweɪ4/ /xoʊ4/ /ʈʂʰu1/ /ʈʂʰɑŋ3/ |
| 3 | /ʐɑŋ4/ /tʰa1/ /pɑŋ1/ /ɕja4/ /li3/ /mu4/ |
| 4 | /mɪŋ2/ /tʰjɛn1/ /i4/ /tɕʰi3/ /tɕi1/ /fan4/ |
| 5 | /pa1/ /tjɛn3/ /kʰɤ3/ /i3/ /ʈʂʰu1/ /fa1/ |
| 6 | /a1/ /li4/ /tɕɪn1/ /tʰjɛn1/ /pu4/ /nɤŋ2/ /ʈʂʰu1/ /ɥœn4/ |
| 7 | /ɕja4/ /u3/ /ta4/ /kaɪ4/ /i1/ /tjɛn3/ /tɕi2/ /xɤ2/ |
| 8 | /tɕʰɥœn2/ /tʰi3/ /tɕɪŋ3/ /ʈʂʰa2/ /pi4/ /ɕy1/ /tɑʊ4/ /ʈʂʰɑŋ3/ |
| 9 | /tuɔ1/ /tɕi1/ /ʂu1/ /tsʰaɪ4/ /ʂɑʊ3/ /tɕi1/ /ʈʂu1/ /ʐoʊ4/ |
| 10 | /ɕi3/ /i1/ /jɛ4/ /pu4/ /nɤŋ2/ /ʈʂʰɑʊ1/ /kuɔ4/ /pa1/ /xɑʊ2/ /ʂɤŋ1/ |
| 11 | /tɕʰɪŋ1/ /ʂɑʊ4/ /njɛn2/ /kʰɤ3/ /i3/ /ʂɚ4/ /tɑŋ4/ /wan2/ /joʊ2/ /ɕi4/ |
| 12 | /tɕʰɥœn2/ /tʰi3/ /ɥœn2/ /kʊŋ1/ /toʊ1/ /jɑʊ4/ /tsʰan1/ /tɕja1/ /pʰeɪ2/ /ɕyn4/ |
| 13 | /kʰaɪ1/ /xweɪ4/ /tɕʰi1/ /tɕjɛn1/ /pu4/ /nɤŋ2/ /na2/ /ʈʂʰu1/ /ʂoʊ3/ /tɕi1/ |
| 14 | /ma2/ /la4/ /tʰɑŋ4/ /tsaɪ4/ /pa1/ /i1/ /lu4/ /xɤ2/ /ʂuən4/ /i4/ /lu4/ /tɕjɑʊ1/ /ʈʂʰa1/ /kʰoʊ3/ |
| 15 | /uɔ3/ /meɪ2/ /uən4/ /tʰi2/ |
| 16 | /ɕjɑʊ3/ /li4/ /meɪ2/ /laɪ2/ |
| 17 | /tsaɪ4/ /pu2/ /tɕʰy4/ /tɕjoʊ4/ /wan3/ /lɤ0/ |
| 18 | /uɔ3/ /u3/ /tjɛn3/ /jɑʊ4/ /tɕʰy4/ /ta4/ /ljɛn2/ |
| 19 | /uɔ3/ /ɕja4/ /u3/ /tɕʰy4/ /na2/ /kʰuaɪ4/ /ti4/ |
| 20 | /tʰa1/ /tsweɪ4/ /tɕɪn4/ /tɕɪŋ1/ /ʈʂʰɑŋ2/ /pu2/ /tsaɪ4/ |
| 21 | /lɑʊ3/ /u2/ /pi3/ /saɪ4/ /joʊ4/ /ɪŋ2/ /lɤ0/ |
| 22 | /na4/ /ni3/ /kʰɤ3/ /i3/ /tɕʰy4/ /ʈʂɑʊ3/ /ta4/ /kɤ1/ |
| 23 | /u3/ /ʂu1/ /pu4/ /kʰɤ3/ /nɤŋ2/ /pu4/ /laɪ2/ /tɤ0/ |
| 24 | /tʰa1/ /ʈʂɤ4/ /tsʰɯ4/ /ʈʂʰu1/ /ʈʂʰa4/ /meɪ2/ /taɪ4/ /uɔ3/ |
| 25 | /a1/ /li4/ /tɕjoʊ4/ /ɕi3/ /xwan1/ /tɕi1/ /ta4/ /ʈʂa2/ /ɕjɛ4/ |
| 26 | /ʈʂɤ4/ /ɕja4/ /uɔ3/ /kʰɤ3/ /i3/ /xwan4/ /ʂoʊ3/ /tɕi1/ /lɤ0/ |
